# Biological Safety Assessments of High-Purified Ovine Collagen Type I Biomatrix for Future Therapeutic Product: International Organisation for Standardisation (ISO) and Good Laboratory Practice (GLP) Settings

**DOI:** 10.3390/polym15112436

**Published:** 2023-05-24

**Authors:** Nur Izzah Md Fadilah, Nazeha Ahmat, Looi Qi Hao, Manira Maarof, Nor Fadilah Rajab, Ruszymah Binti Hj Idrus, Mh Busra Fauzi

**Affiliations:** 1 Centre for Tissue Engineering and Regenerative Medicine, Faculty of Medicine, Universiti Kebangsaan Malaysia, Kuala Lumpur 56000, Malaysia; izzahfadilah@ukm.edu.my (N.I.M.F.); nazehaahmat@ukm.edu.my (N.A.); looi_daniel@yahoo.com (L.Q.H.); manira@ppukm.ukm.edu.my (M.M.); ruszyidrus@gmail.com (R.B.H.I.); 2 My Cytohealth Sdn. Bhd., Kuala Lumpur 56000, Malaysia; 3 Biomedical Science Program, Center for Healthy Aging and Wellness, Faculty of Health Sciences, Universiti Kebangsaan Malaysia, Jalan Raja Muda Abd Aziz, Kuala Lumpur 50300, Malaysia; nfadilah@ukm.edu.my

**Keywords:** collagen, ovine tendon, biomatrix, compatibility, toxicity, wound healing

## Abstract

Wound care management is incredibly challenging for chronic injuries, despite the availability of various types of wound care products in the market. However, most current wound-healing products do not attempt to mimic the extracellular matrix (ECM) and simply provide a barrier function or wound covering. Collagen is a natural polymer that involves a major constituent of the ECM protein, thus making it attractive to be used in skin tissue regeneration during wound healing. This study aimed to validate the biological safety assessments of ovine tendon collagen type-I (OTC-I) in the accredited laboratory under ISO and GLP settings. It is important to ensure that the biomatrix will not stimulate the immune system to produce any adverse reaction. Therefore, we successfully extracted collagen type-I from the ovine tendon (OTC- I) using a method of low-concentration acetic acid. The three-dimensional (3D) skin patch of spongy OTC-I was a soft and white colour, being tested for safety and biocompatibility evaluations based on ISO 10993-5, ISO 10993-10, ISO 10993-11, ISO 10993-23, USP 40 <151>, and OECD 471. For the dermal sensitisation and acute irritation test, none of the tested animals displayed any erythema or oedema effects (*p* > 0.005). In addition, there were no abnormalities detected in the organ of the mice after being exposed to OTC-I; additionally, no morbidity and mortality were observed in the acute systemic test under the guideline of ISO 10993-11:2017. The grade 0 (non-reactive) based on ISO 10993-5:2009 was graded for the OTC-I at 100% concentration and the mean number of the revertant colonies did not exceed 2-fold of the 0.9% *w*/*v* sodium chloride compared to the tester strains of *S. typhimurium* (TA100, TA1535, TA98, TA1537), and *E. coli* (WP2 *trp uvrA*). Our study revealed that OTC-I biomatrix does not present any adverse effects or abnormalities in the present study’s condition of induced skin sensitization effect, mutagenic and cytotoxic towards cells and animals. This biocompatibility assessment demonstrated a good agreement between in vitro and in vivo results regarding the absence of skin irritation and sensitization potential. Therefore, OTC-I biomatrix is a potential medical device candidate for future clinical trials focusing on wound care management.

## 1. Introduction

Wound healing represents a substantial financial burden in current healthcare systems with estimated annual healthcare expenditures. Malaysia’s advanced wound care market size was valued at USD 47.63 million in 2021 and is projected to reach USD 117.31 million by 2030, growing at a compound annual growth rate (CAGR) of 12.17% from 2022 to 2030 [1]. Statistics from the Malaysian Ministry of Health (MOH) showed that out of the over 3 million diabetic Malaysia citizens, 15% will develop lower extremity ulcers [2]. It is an important and complex process, which normally involves three successive but overlapping phases, including haemostasis/inflammatory phase, proliferative phase, and remodelling phase [3,4]. Clotting happens because of direct contact between endothelial cells and platelets and the initiation of the coagulation cascade after an injury. In the early process, mediators cause an inflammatory response that draws neutrophils and macrophages from the circulation. The recruitment of stromal cells and their differentiation into myofibroblasts, which are in charge of wound contraction and extracellular matrix (ECM) deposition, is brought on by these cells’ production of proinflammatory cytokines and growth factors. In order to promote angiogenesis and re-epithelialisation, respectively, they can boost endothelial and epithelial cell proliferation in the wound site [5,6].

Chronic wounds include a wide variety of aetiologies and intricate relationships between risk factors. Therefore, there is a critical need for trustworthy, proven risk prediction methods that can more effectively target each patient’s particular set of predisposing factors, which affect each patient’s likelihood of disease development and treatment response once chronic wounds have formed [7]. One of them is burns, an injury that can cause wounds, infection, ulcers, and trauma; however, those caused by chronic illnesses such as diabetes, vascular diseases, and cancers can lead to delay and low wound-healing progress [8]. The burn injury that would be associated with significant morbidity or mortality involves immune reaction, metabolic alterations, and distribution shock that is difficult to manage. In a serious case, burn injuries may be threatening to life when there is an infection involved. The area that was loss in skin cell will provide higher chances for microorganisms such as bacteria and other pathogens to invade the body since the first layer of protection was absent [9].

These problems related to treating wounds are snowballing with the increasing health care costs and an incidence of an aging population. It is necessary and significant to call for a modernisation of wound management methods that are effective and safe for accelerating wound healing. Generally, the healing of wounds requires interactions between cell and tissue components of the surrounding damaged skin, which includes the dermal and epidermal cells (fibroblast and keratinocytes), ECM, and the nervous and vascular components [10,11]. Hence, a research hotspot in the tissue engineering and regenerative medicine (TERM) field is how to assist wound healing with high efficacy, mainly in advanced functional biomaterials technology.

Clinically, the current gold standard for full-thickness skin loss is split-thickness autologous skin grafting [12]. The way split-skin graft works is epidermal, with a superficial part of the dermis being harvested with dermatome from an undamaged skin donor site and then applied to a full-thickness wound. In the wound site, the split-skin graft (SSG) capillaries form anastomoses into the existing capillary network to provide nutrients for graft survival [13]. Nevertheless, it has major drawbacks as the procedure is limited to donor skin, exposed to microbial infection and has no resemblance to tissue regeneration [14]. Therefore, tissue engineering was used as an alternative treatment to repair and regenerate living tissue with compositions, structures, and functions comparable to native tissue via different approaches used in multiple disciplines and fields, including engineering, biology, and medicine [15,16,17]. The worst-case scenarios are severe infection and secondary bacteraemia that would impede wound healing and leg amputation treatment, debilitating the patient’s quality of life and ability to work, increasing the burden on managing diabetic wounds, contributing to depressing socioeconomic status and increasing morbidity. According to the market analysis report carried out by Grand View Research, the global collagen market demand was estimated at 920.1 tons in 2019, and it is estimated to develop at a CAGR of 10.2% from 2023 to 2030. Increasing end-use applications in the healthcare industry, such as bone and joint health supplements, skin wound dressing, tissue regeneration, medical implants, cardiology, and drug delivery, collagen has been found to be one of the most used biomaterials [18].

Alternatively, collagen is the most abundant ECM protein where collagen type-I supramolecular assemblies, such as tissue grafts, and cell-assembled scaffolds, which are used extensively in TERM [19]. It is also considered the biological fixative that holds cells and vascular tissues in place. The modern view considers collagen the primary ECM molecule that self-assembles into cross-striated fibrils, responsible for supporting cell growth and the mechanical resilience of connective tissues. In addition, collagen is found in a proportion of about 70% to 80% at the skin level, present in all dermal dry matters. The interactions between collagen and cells are essential during wound healing because collagen promotes the maintenance and differentiation of cellular phenotypes [20]. Interestingly, thousands of studies reported using collagen as an excellent candidate for texturizing, thickening, and gel formation due to its high water absorption capacity [21,22]. Moreover, collagen possesses desirable biocompatibility and biodegradability, low immunogenicity, good shape consistency at 37 °C (human body temperature), and excellent microstructure of micro- and macropores that promote cellular attachment and proliferation [23]. Collagen type-I can be extracted from different tissues such as tendon, ligament, muscle, skin, bone, and from the intestine of various species including ovine, caprine, bovine, porcine, and marine sources using a controlled series of biological processes [24,25]. Collagen comprises over 80% of the dry weight in the tendon and forms a hierarchically structured fibrillar network aligned predominantly along the direction of loading.

Among many available extraction methods, we developed a new approach to extract collagen type-I from ovine tendon collagen (OTC-I) using a low concentration of acetic acid [26,27,28. The extracted collagen using this protocol was proven to possess similar physicochemical properties as many commercially available collagen type-I. The proof of concept from the previous studies has been published elsewhere to demonstrate the capability of OTC-1 use for acellular skin substitute [26,29,30,31]. Some previous studies have reported successfully extracted collagen from ovine tendon and proven it contains a high yield of collagen type-I through a low concentration of acetic acid, with no cytotoxicity [32,33], no immune response through the in vitro and in vivo evaluation of the pre-clinical model for the efficiency study. Consequently, this study aimed to evaluate the safety and biocompatibility testing of OTC-I via in vitro and in vivo assessments. We evaluated the potential of OTC-I to produce irritation via intradermal injection and to assess its potential to cause a delayed hypersensitivity reaction following exposure on the skin of guinea pig. In addition to that, the pyrogenic effect of OTC-I was determined by measuring a rise in temperature following an intravenous injection in rabbits. In addition, intravenously injection into the mice’s tails to observe the toxicity evaluation via systemic intervention was also conducted in this study. A further cytotoxicity effect of OTC-I was observed using mammalian cell line (L-929 cell line) and its mutagenicity effects via bacterial reverse mutation.

## 2. Materials and Methods

This study was approved by the UKM Animal Ethics Committee (BIOSERASI/UKM/2022/MIMI NORHILDA/18-FEB./1232-FEB.-2022-FEB.-2023). All experiments have been performed by Biocompatibility Laboratory, Universiti Kebangsaan Malaysia as required by Medical Device Authority (MDA), Ministry of Health (MOH) Malaysia.

### 2.1. Extraction and Purification of Collagen Type-I from Ovine Tendon

The extraction process was performed as described previously by Fauzi et al. [26]. Briefly, the raw ovine tendon was washed and scrubbed, through which all the fascia and muscle tissues were removed. The clean tendon was cut into small pieces and dissolved in 0.35 M acetic acid (Merck, Darmstadt, Germany) at 4 °C for 24–48 h. The tendon with the acetic acid solution was blended prior to centrifugation at 5000 rpm at 4 °C for 5 min. The impurities in the pellet form were removed; meanwhile, the supernatant containing collagen was collected and neutralised to pH 7 by using 1 M sodium hydroxide, NaOH (Sigma-Aldrich, St. Louis, MO, USA). The collagen solution was then added into a dialysis tube (molecular weight cut-off 14 kDa; Sigma-Aldrich, St. Louis, MO, USA) and incubated at 4 °C for 72 h with alternate changes of chilled distilled water every 12 h. After that, the clear solutions on top of the tube were removed and the remaining contents (collagen solution) were pre-frozen at −80 °C for 6 h followed by freeze-drying (Ilshin, Korea) for 48–72 h. The dried collagen was redissolved in 0.35 M acetic acid and blended again to prepare a collagen stock solution with a final concentration of 14 mg/mL before being kept at 4 °C for further use in the next experiment.

### 2.2. Fabrication of Collagen Sponge (OTC-I)

The collagen sponge, referred to as OTC-I, was prepared with the desired mould by using collagen solution from the final stock. The OTC-I was fabricated by pre-freezing the collagen solution at −80 °C for 6 h, followed by freeze-drying it for 72 h at −56 °C and 5 mTorr of atmospheric pressure to form a collagen sponge.

### 2.3. Skin Sensitisation Test (Guinea Pig Maximisation Test)

#### 2.3.1. Animals Monitoring

The purpose of a skin sensitisation test is to evaluate the potential of OTC-I to cause a delayed hypersensitivity reaction (Type IV) following exposure to the skin of guinea pig. A total number of 25 Dunkin Hartley albino guinea pig with an average body weight of 390–490 g were tested in this study according to their groups including OTC-I group (10 males), negative control group (5 females), and positive control group (total 10; 7 males and 3 females). The control items are normal saline as negative control; meanwhile, 0.08% of 1-Chloro-2,4-Dinitrobenzene (DNCB) in 80% ethyl alcohol acts as positive control. The guinea pigs were housed in appropriate suspended stainless-steel cages with mesh floors as recommended in Animal Husbandry Guideline. They were fed with a standard diet of pellet and fresh vegetables and filtered tap water ad libitum; a total of 6 animals were placed per cage at a room environment temperature of 20–23 °C and 12 h of artificial and natural light (alternating) with dark cycle at Laboratory Animal Resource Unit, UKM and Centre for Research and Instrumentation Management (CRIM). The identification card was attached to each cage and each guinea pig was marked with a colour code (permanent marker pen) on the forehead and given a sequential animal number assigned to a job number, which constitutes a unique identification system.

#### 2.3.2. Pre-Dosing Procedures

Following the pre-dosing procedures, the guinea pigs were checked for ill health and only healthy animals were selected to be used in the study. They oriented themselves within five days (acclimation step). Their cages were labelled with an animal identification card and all animals were weighed prior to the treatment.

#### 2.3.3. Preparation of the Skin

The shoulder region of each guinea pig was clipped free of hair and exposed in an area on the shoulder region measuring 4 cm by 6 cm within 24 h prior of initiating treatment. After the shaving process, the skin of each guinea pig was examined for any abnormalities and ill health. Only animals without pre-existing skin lesions on the test sites were selected.

#### 2.3.4. Preparation of Test and Control Items

There are three types of preparation for the intradermal injection test. First, an equal ratio of Freund’s complete adjuvant and normal saline solution was mixed to form a stable emulsion. Second, the OTC-I was extracted in normal saline (polar solvent) with an extraction ratio of 3 cm^2^/mL and temperature at 37 ± 1 °C for 72 ± 2 h in a water bath shaker. Third, all the OTC-I extracts, negative or positive control items were prepared by mixing at 50:50 volume with a stable emulsion of Freund’s complete adjuvant. Meanwhile, as for topical application, the test item was prepared by applying 0.5 mL of OTC-I extract on filter papers, which measured 2.0 × 4.0 cm and 2.0 × 2.0 cm for the induction phase and the challenge phase, respectively.

#### 2.3.5. Application of Test Items

This study was conducted in accordance with the standard guideline of ISO 10993-10 [34]. The OTC-I extracts and controls (0.1 mL per site) were then applied on three pairs of injection sites, which are designated as A, B, and C, at the intra-scapular region of the guinea pigs, as shown in Table 1.

**Figure 1:**
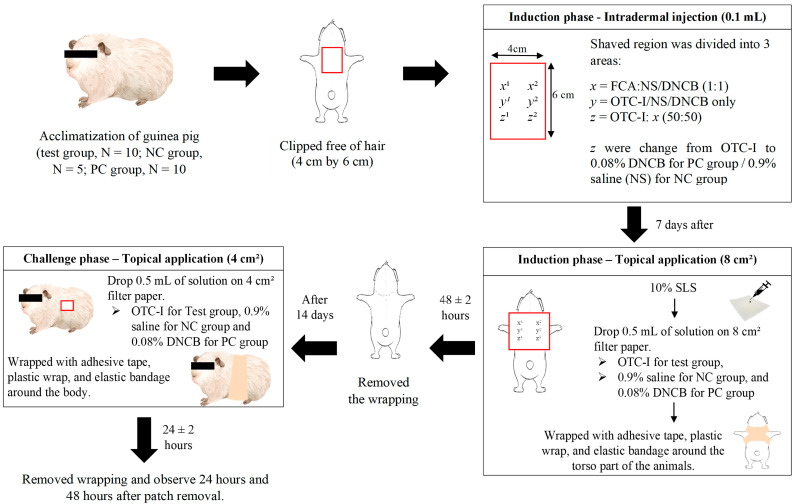
Experimental flow for guinea pig maximisation test.

The application of test items is topically applied within two phases, the induction and the challenge phases. After completion of the intradermal induction (7 days) during the induction phase, the OTC-I extract, negative, and positive control were topically applied on the same injection sites using 8 cm^2^ filter paper and absorbent gauze. The patching area was pre-treated with 10% sodium lauryl sulphate in petroleum jelly for 24 ± 2 h before the patch was applied. Following that, the filter paper was covered with gauze and adhesive tape, and the patch was then wrapped with plastic wrap and an elastic bandage (occlusive dressing) around the torso to secure the test item for 48 h. After 14 days, due to completion of the topical induction phase, the OTC-I extracts were patched onto the untreated skin of guinea pigs for 24 ± 2 h. The patches were then removed, and the skin was examined for skin reactions at 24 ± 2 h and 48 ± 2 h. Each site of skin was graded for erythema and oedema in accordance with the description in Table 2. Figure 1 shows an experimental flow for guinea pig maximisation test that was carried out in the study.

### 2.4. Rabbit Pyrogen Test

#### 2.4.1. Animals Monitoring

The pyrogen test aims to determine the pyrogenicity effect of test item (OTC-I) by measuring a rise in temperature following an intravenous injection in rabbits. There are three white female rabbit’s species in which New Zealand animal strain. The rabbits were housed in appropriate suspended stainless-steel cages with mesh floors as recommended by Animal Husbandry Guideline. All the animals were selected randomly prior to acclimation. Each cage included only one animal and was attached with an identification card labelled with an animal number, gender, and study reference number.

#### 2.4.2. Pre-Dosing Procedures

On arrival and just before dosing, all the animals were weighed before treatment and checked for health status. Thus, only healthy animals were selected and used in the study.

#### 2.4.3. Administering of Test Solution

The ear of each rabbit was clipped free of hair with a clipper and wiped with an alcohol swab. Prior to the administering of the test solution, two tests were evaluated. First, using the Sham test, where the rabbits’ body temperatures were measured three times at 30 min intervals. Second, the main test, where OTC-I samples were extracted individually in 40 mL of normal saline (0.9% sodium chloride) at 37 ± 1 °C in a shaking water bath for 72 ± 2 h. Then, the extraction was pooled for intravenous injection. The test item was injected at 10 mL/kg of body weight. The temperature of rabbit’s body was measured within 30 min prior to injection of the test item and six times every 30 min within 3 h after injection of the test item as recommended in USP 40 <151> [35].

### 2.5. Intracutaneous (Intradermal) Animal Irritation Test

#### 2.5.1. Animals Monitoring

The purpose of this experiment is to assess the potential of a test item (OTC-I) extract to produce irritation following intradermal injection of test item extract. In this study, all female white rabbits (New Zealand animal strain) were used as test groups. The rabbits were handled through the same procedures as in Section 2.4.1, as the study procedure followed ISO 10993-23 [36].

#### 2.5.2. Pre-Dosing Procedures

The rabbits were checked for ill health and weighted following the same procedures as in the previous section.

#### 2.5.3. Preparation of the Skin

In this study, only animals without pre-existing skin irritation were selected. The dorsal surface of each side of the rabbit was clipped free of hair with a clipper to expose a surface, allowing a sufficient distance to inject the test item extract. Better care was taken to avoid abrading the skin and trauma. After exposing the skin and before treatment, animals were again examined for abnormalities and ill health.

#### 2.5.4. Preparation of Test and Control Items

The test (OTC-I) extract with a surface area of 14.139 cm^2^ piece was extracted in two different solvents, which are polar solvent (normal saline) and non-polar solvent (cottonseed oil) at 37 ± 1 °C for 72 ± 2 h using a 3 cm^2^/mL extraction ratio.

#### 2.5.5. Application of Test Items

The test (OTC-I) extract and control items were applied to each designated site. Each rabbit was administered with 0.2 mL of the test item extracted in normal saline and 0.2 mL of normal saline at five anterior sites, respectively, via intradermal injection. The steps were repeated on the other side of the animal using the extract obtained with non-polar solvent and its non-polar solvent control (cottonseed oil). Before the observations, the skin lesions on each application site were evaluated immediately after injection and at 24 ± 2 h, 48 ± 2 h, and 72 ± 2 h post-injection according to the scoring system as shown in Table 3. The test complies with ISO 10993-23:2021 (E) [36].

### 2.6. Acute Systemic Toxicity Study (Intravenous)

#### 2.6.1. Preparation of Mice

In total, 10 female Balb/c mice (specific pathogen free) aged around 9–10 weeks old were used in this test. All the mice were nulliparous, non-pregnant and housed in a controlled environment at 19 °C to 25 °C, with a relatively high humidity of 30–70% and an artificial lighting sequence of 12 h light and 12 h dark. The mice were fed a Altromin 1324 maintenance diet for rats and mice and filtered drinking water ad libitum. Each animal was housed in appropriate polycarbonate cages with solid bottoms and sides with stainless steel lids. Mice that were healthy after 7 days of acclimation and had a weight range not more than ± 20% of the mean body weight were chosen to be used in this test.

#### 2.6.2. Preparation of the OTC-I

The OTC-I was extracted with an extraction ratio of 3 cm^2^/mL as recommended in ISO 10993-12:2012 Biological Evaluation of Medical Devices—Part 12: Sample preparation and references materials by using 0.9% normal saline [37]. The control group (normal saline) was required for each mouse and measured based on the dosage volume 50 mL/kg of body weight. The temperature of 37 ± 1 °C was maintained for 72 ± 2 h in a shaking water bath while the test item was extracted.

#### 2.6.3. Exposure Procedure

The test was conducted in accordance with ISO 10993-11:2017(E). Biological evaluation of medical devices—Part 11: Tests for systemic toxicity [38]. Single dose of OTC-I and 0.9% normal saline were injected intravenously into the group, respectively, for the first time and followed with multiple fraction doses within 24 h duration. Mice were restrained prior to dosing and the tail vein as an injection site was prepared by dipping in warm water at approximately 30–35 °C for at least 1 min to stimulate the dilation. The mice were weighed on day 0, 1, 2 and 3 before euthanising the animal. The behaviour of each animal was evaluated based on Table 4.

### 2.7. MEM Elution Assay Using L-929 Cell Line

The purpose of MEM elution assay was to assess the cytotoxicity effect of test item via a qualitative morphology observation using mammalian cell line (L-929 cell line).

#### 2.7.1. Preparation of L-929 Cell Line

As recommended in the ISO 10993-5:2009 guideline [39], cell line L-929 (NCTC clone 929) was used in this study as a test system, obtained from American Type Culture Collection (ATCC) (ATCC No. CCL-1™). The cell line was only used after testing with mycoplasma’s absence. The cells were cultured as a monolayer in a culture medium with serum in 37 ± 1 °C at 5 ± 1% carbon dioxide and 95% air. Once the confluent reached more than 90%, the cell was trypsinised and the number of viable cells was calculated. By using the 24-well plate, seed the cell at the seeding density of 1.0 × 10⁵ cells/well and incubated at 37 ± 1 °C for at least 12 h or until attaining 70–80% confluency.

#### 2.7.2. Preparation of OTC-I

The sterile OTC-I was handled aseptically in the biological safety cabinet. The OTC-I was incubated for 24 ± 2 h at 37 ± 1 °C in a shaking water bath at a 3 cm^2^/mL extraction ratio. The extracted solution was considered 100% extraction and directly used in this test.

#### 2.7.3. Preparation of Control Item

Polyethylene (negative control), zinc diethydithiocarbamate (positive control) and the minimum essential medium (MEM) enriched with serum (as blank) were prepared in the same environment of extraction, the conditions of which were at 37 ± 1 °C in the shaking water bath for 24 ± 2 h. Negative control and positive control were extracted in a culture medium with serum at the extraction ratio of 0.2 g/mL. Prior to usage in the test, negative and positive control were sterilized by filtering these using 0.2 μm membrane while the blank was directly used after the 24 ± 2 h of extraction.

#### 2.7.4. Exposure Procedure

At a concentration of 100%, the test item extract was tested in triplicate. Each well of a 24-well plate containing a healthy culture had its growth medium replaced with 1 mL of the test item extract, negative control, positive control, and blank, respectively. The cultures were then incubated for 24 ± 2 h at 37 ± 1 °C in a humidified atmosphere containing 5% CO_2_ and 95% air. After 24 ± 2 h of incubation, the cell behaviours in each well were evaluated microscopically and scored from 0 to 4 according to ISO 10993-5:2009 Biological evaluation of medical devices—Part 5: Tests for in vitro cytotoxicity [39] as listed in Table 5.

### 2.8. Bacterial Reverse Mutation Test Using S. typhimurium and E. coli

The bacterial reverse mutation assay (Ames test) provides a rapid assessment of the mutagenic potential of a compound to cause mutations in the deoxyribonucleic acid (DNA) of the test organism [40]. The method employs amino acid requiring strains of *Salmonella typhimurium (S. typhimurium)* and *Escherichia coli (E. coli)* to detect mutations. The basic idea behind this assay is that it detects mutations that revert mutations present in the test strain and restore the bacteria’s functional ability to synthesis an essential amino acid. The ability of revertant bacteria to grow without amino acids is used to identify them. The study was carried out in two different manners: with and without metabolic activation as written in OECD 471 [41].

#### 2.8.1. Preparation of Bacterial Strains

The four strains of *S. typhimurium* (Strain TA98, Strain TA100, Strain TA1535 and Strain TA1537) and one strain of *E. coli* named WP2 *trp uvrA* that were used in this study were purchased from Molecular Toxicology Inc. (MOLTOX). The strains that obtain histidine for *S. typhimurium* and tryptophan for *E. coli* were the only accepted to be used and stored in liquid nitrogen. After thawing frozen bacterial culture stock, 12 μL of the culture was suspended in 12 mL of nutrient broth. The broth was then incubated for approximately 7 ± 2 h in a refrigerator, followed by incubation in a shaking water bath at 37 ± 2 °C until the culture reached the late exponential or early stationary phase of culture growth. By using a spectrophotometer, the optical density (OD) of bacterial suspension was determined at 660 nm.

#### 2.8.2. Preparation of OTC-I

The ISO 10993-12:2021 (E, Biological evaluation of medical devices—Part 12: Sample preparation and reference materials) was used to prepare the OTC-I to be used in this study [37]. Since the OTC-I was sterile, it was handled aseptically in the biological safety cabinet. The OTC-I was extracted in normal saline (0.9% *w*/*v* sodium chloride) at a 3 cm^2^/mL extraction ratio in a shaking water bath for 72 ± 2 h at 37 ± 1 °C. The 100% OTC-I extract was diluted with normal saline (0.9% *w*/*v* sodium chloride) into four different concentrations with approximately half-log intervals. This test employed five concentrations of OTC-I (6.25%, 12.5%, 25%, 50%, and 100%).

#### 2.8.3. Preparation of Control Item

Negative control for the whole study was 0.9% *w*/*v* sodium chloride (normal saline). The normal saline was extracted in the same condition as OTC-I, which was incubated in a 37 ± 1 °C shaking water bath for 72 ± 2 h. Positive control stock for this study was freshly diluted using normal saline and prepared into final concentration as in Table 6.

#### 2.8.4. Exposure Procedure

Each assay was performed in triplicate using the pre-incubation method. In a test tube, 0.5 mL of sterile phosphate buffer, 0.1 mL of OTC-I or control item, and 0.1 mL of bacteria culture were mixed for the assay without metabolic activation. In the test tube for the assay with metabolic activation, 0.5 mL of S9 mix (5% *v*/*v* S9 fraction in cofactor), 0.1 mL of test item or control, and 0.1 mL of bacteria culture were mixed. The test tubes were then pre-incubated in a shaking water bath for 20–30 min at 30–37 °C. After the incubation period, 10% of 0.5 mM of histidine (for tube with strain *S. typhimurium*) or 0.5 mM tryptophan (for *E. coli*) and 2 mL of the enriched top agar were added to the tube. The content of each test tube was mixed and evenly distributed onto the surface of glucose minimal agar plates. The overlay agar was allowed to solidify before being incubated at 37 ± 2 °C for 48 ± 2 h.

## 3. Results

### 3.1. Gross Appearance of OTC-I

**Figure 2:**
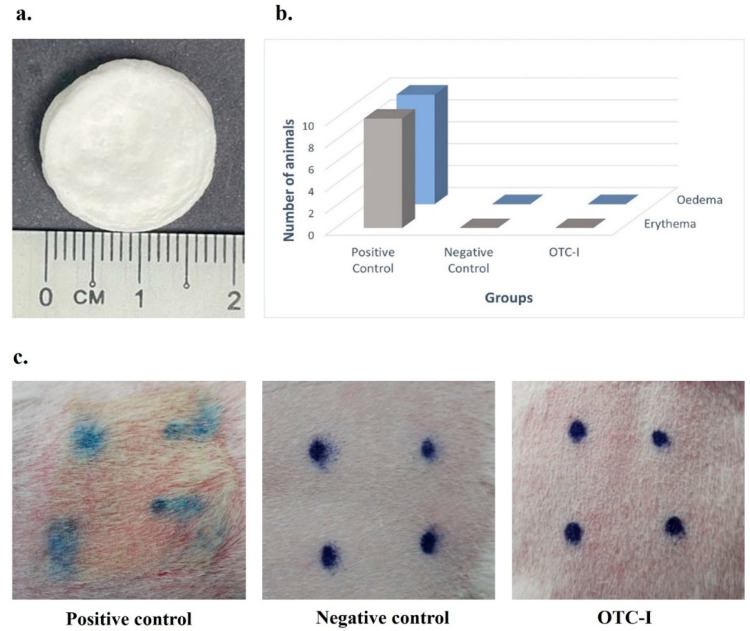
(**a**) Gross appearance of OTC-I scaffold, (**b**) response indices up to 48 h after patch removal during the challenge phase, and (**c**) sensitisation effect on guinea pig skin after patch removal.

The gross appearance of OTC-I scaffold is illustrated in Figure 2a. The scaffold was in sponge form and appeared in white. Fauzi et al. produced OTC-I through acid-solubilised protein extracted from the ovine tendon using 0.35 M acetic acid [26]. They found that 80% of extracted protein was measured as collagen. The results indicated that collagen type-I was the most abundant protein in the OTC solution. The chemical characterisation of OTC-I was performed via Fourier transform infrared spectrometry (FTIR), X-ray diffraction (XRD) and energy dispersive x-ray spectroscopy (EDS) in comparison with rat tail Col I (control). The previous studies reported all the results for physicochemical properties and micrograph structures [26,42].

### 3.2. OTC-I Demonstrates No Sensitisation Effect via Guinea Pig Maximisation Test

After induction and challenging step, no sensitisation effects were produced on the skin of guinea pigs following patch removal with observations up to 48 h, as presented in Figure 2b. The polar solvent extract of OTC-I did not induce a skin sensitisation effect in the guinea pigs (grade 0). Both the negative and positive control groups performed as anticipated. However, the positive control animals exhibited moderate (grade 3), confluent erythema, and intense erythema and/or swelling. The photos’ evaluation of in vivo immune response via guinea pig maximisation test of OTC-I scaffolds is presented in Figure 2c.

### 3.3. OTC-I Displays the Absence of Pyrogenic Substances via the Rabbit Pyrogen Test

The body temperature was measured, recorded, and tabulated beginning within 30 min prior to injection of the test item and up to 30 h following injection at 30 min intervals. The differences between the control temperature reading and the maximum temperature recorded are taken as the response. Results of the Sham and Main tests showed that all three rabbits did not exceed the 0.5 °C of control temperature limit, as presented in Table 7 and Table 8, respectively. Thus, the test item of OTC-I meets the requirement for the absence of pyrogenic substances.

### 3.4. OTC-I Did Not Demonstrate a Potential to Produce Irritation via Animal Irritation Test

By observing the animals, all three rabbits appeared active and healthy with no signs of gross toxicity, adverse pharmacologic effects, or abnormal behaviour. There was no sign of erythema and oedema noted on all sites injected with the test item extracted in normal saline as polar solvent control on all rabbits at 24 ± 2, 48 ± 2, and 72 ± 2 h post-injection. However, on the sites injected with the test item extracted in cottonseed oil, very slight erythema (score 1) was noted on one site of rabbit 1 at 24 ± 2 h post-injection and progressed to well-defined erythema (score 2) on two sites at 48 ± 2 and 72 ± 2 h observation. Additionally, very slight erythema (score 1) was noted on one site at non-polar solvent control sites at 24 ± 2 h post-injection and throughout the study period. For rabbit 2, very slight erythema (score 1) was observed on one site at 48 ± 2 h post-injection but regressed to no erythema at 72 ± 2 h of observation. Another result for rabbit 3 whereby a very slight erythema (score 1) was examined at one site at 24 ± 2 h post-injection and throughout the study period. The difference in the mean scores between the test item extracted in normal saline and polar solvent control was ‘0′, and that of the test item extracted in cottonseed oil and non-polar solvent control was ‘0.22′. The skin reaction scores of the test item and controls are presented in Table 9. Since the difference between polar and non-polar of test item extract and their respective control mean scores was less than 1.0, the test item extract of OTC-I did not demonstrate potential to produce irritation under the conditions of this study.

### 3.5. OTC-I Reveals No Adverse Effects via Acute Systemic Toxicity Study (Intravenous)

**Figure 3:**
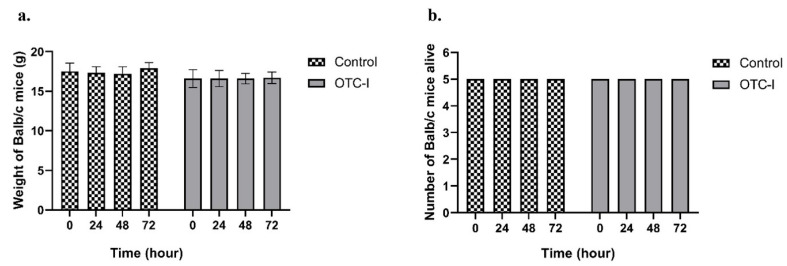
(**a**) Body weight of Balb/c mice, and (**b**) mortality rate of each group before injection (0 h) and after day 3 throughout the experimental time. The results were not significantly different (*p* > 0.05) between the groups.

The mice were observed before the intravenous injection was applied and recorded as 0 h showing that the mice were healthy and did not exceed the ±20 g from the mean of the group. This random selection showed that the animal has a body weight of 15.9–18.7 g (mean of the body weight was 17.1 g). Prior to euthanasia at 72 ± 2 h, the Balb/c mice were weighed, and any weight loss was recorded by comparing the body weight at 0 to that at 72 h. Figure 3a and b shows the body weight and mortality of the tested animals, respectively.

The signs of toxicity and behavioural changes were also observed and recorded as displayed in Table 10, immediately after injection, 4 ± 1 h after injection until day 3 (72 ± 2 h). The results show that the OTC-I and negative control group were recorded as no abnormalities detected (NAD). After 72 ± 2 h of injection, all the Balb/c mice were euthanised using the overdose carbon dioxide (CO^2^) and the gross necropsy was performed immediately, including the examination of external surface of the body, all orifices, and the cranial, thoracic and abdominal cavities and their contents. The data were tabulated in Table 11 for necropsy findings of Balb/c mice among the group.

### 3.6. OTC-I Shows No Reactivity through MEM Elution Assay Using L-929 Cell Line

**Figure 4:**
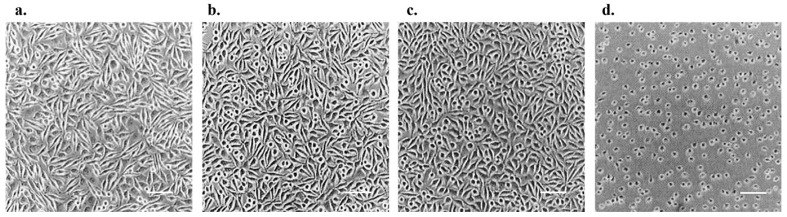
Illustrates the condition of the cells in each well microscopically after 24 ± 2 h of incubation; (**a**) 100% concentration of OTC-I, (**b**) blank (minimum essential medium enriched with serum, (**c**) negative control (polyethylene) and (**d**) positive control (zinc diethyldithiocarbamate). All the microscopic photos are in 4× magnification (bar scale: 100 µm).

After 24 ± 2 h of incubation, cell line L-929 was observed under a microscope and graded according to the score tabulated in the ISO 10993-5. The result shows that the 100% OTC-I was grade 0 with no reactivity due to its discrete intracytoplasmatic granules, no cell lysis and no reduction in cell growth at 0 h and 24 h after. The culture medium with serum (blank extract) demonstrated no reactivity (grade 0), which indicated no confounding effects due to the extraction vessel, vehicle, and extraction process [43]. Meanwhile, the polyethylene (negative control extract) revealed no reactivity too (grade 0), which points to the background response of the test system. However, the zinc diethyldithiocarbonate (positive control extract) exposed severe reactivity (grade 4), which specified an appropriate test system response. Following that, all three control item groups (blank, negative control and positive control) performed as anticipated, indicating the adequate performance of the test. Consequently, to describe the cytotoxicity assay and under the experimental condition reported, the OTC-I did not demonstrate cytotoxic effect. Table 12 shows the reactivity grades of the extracted OTC-I at 100% concentration and control item. Besides that, Figure 4 illustrates the condition and morphology of the cells in each well microscopically after 24 ±2 h of incubation. As in Figure 3a, the cell morphology was unaffected (did not induce changes in morphology) by the OTC-I extracts in comparison with negative control cells and blank cells, demonstrating that this OTC-I is safe. Meanwhile, in Figure 4d, the cell monolayer was destroyed probably due to the toxic effect of the substances present in the media [44].

### 3.7. OTC-I Did Not Induce Point Mutations via Bacterial Reverse Mutation Test

**Figure 5:**
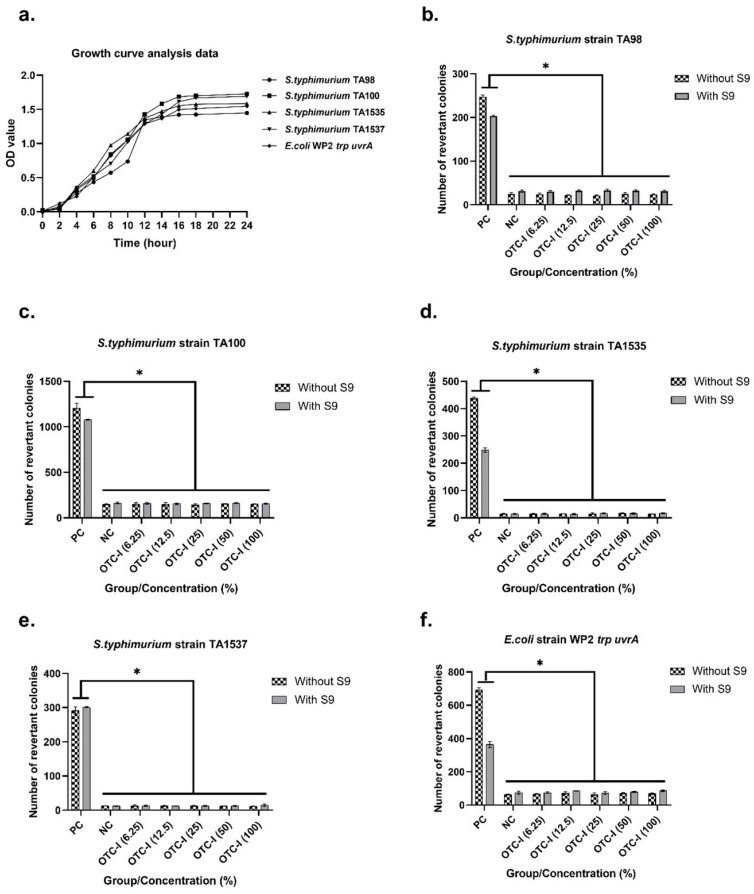
(**a**) Growth curve analysis data and number of revertant colonies of each strain at concentrations of 6.25–100% control without and with metabolic activation (S9) for (**b**) *S. typhimurium* TA98, (**c**) *S. typhimurium* TA100, (**d**) *S. typhimurium* TA1535, (**e**) *S. typhimurium* TA1537, and (**f**) *E. coli* WP2 *trp uvrA*. The * asterisk represents significant difference (*p* < 0.001).

Based on the growth curve analysis data in Figure 5a, all bacterial strains’ late exponential and early stationary phase was between 14 ± 2 h of incubation with shaking. After the incubation time, no precipitation of the test item was seen on any plate. The bacterial background lawn was clearly visible under an inverted microscope for the cytotoxicity evaluation of the OTC-I to the test system, and there was no clearing or diminution of the background lawn in any of the plates. This means that none of the measured concentrations of the test item had a cytotoxic effect on the test system. In addition, the cytotoxicity effect also was determined by any reduction in the number of revertant colonies or the degree of survival of treated cultures. In addition, the cytotoxicity effect also was determined by any reduction in the number of revertant colonies or the degree of survival of treated cultures. The mean number of revertant colonies produced following exposure of the OTC-I in all tested concentrations, as well as those in negative control and positive control, are presented in Figure 5b–f. The results demonstrated that the mean number of revertant colonies of *S. typhimurium* and *E. coli* treated with OTC-I did not exceed by 2-fold of the number of spontaneous revertant colonies in negative control plate, either in the presence or absence of metabolic activation (S9). However, the positive control generated a significant number of revertant colonies.

## 4. Discussion

Biomaterials are substances engineered to engage with biological systems to restore or repair damaged tissue. In TERM field, biomaterials that can be made from various sources, either synthetic or naturally occurring in the origin of the material, such as polymers, ceramics, metal, and composites, play a role by acting as a host for the cells to grow and differentiate into functional tissue. Over the decades, research about functional biomaterial in tissue engineering has been explored worldwide due to its beneficial effect in various applications such as medical, food, and cosmetic applications. The properties of collagen as biomaterials that make them useful in these fields include good biodegradability, excellent biocompatibility, non-toxicity, non-antigenicity and non-immunogenicity, and ready availability [45,46,47]. In the current study, we have fabricated OTC-I in sponge form as a 3D scaffold. The biocompatibility of OTC-I scaffold was tested and the results showed that OTC-I scaffolds facilitate the attachment and proliferation of both skin cells, specifically keratinocyte and fibroblast cells, as has been presented in previous research [29,30]. Following the MDA requirements, all the pre-clinical testing has been carried out in the accredited laboratory. In addition to the safety biocompatibility testing, multiple toxicity endpoints are still demanded for animal experiments, particularly the tests for skin sensitisation where huge amounts of laboratory guinea pigs are used. Based on the immunogenicity testing, the OTC-I scaffold has been proven inert and caused no immune response in vivo. Consequently, it could be considered safe to use in the clinical setting in long-term contact with the human body.

Accordingly, the rabbit pyrogen test is the most employed safety assay used to detect pyrogens [48]. In pyrogenic studies in rabbits, it has been found that the results turn to a zero response, where the difference between the control temperature reading and maximum temperature is negative. Any temperature decreases are considered zero rises. Interestingly, the results demonstrated that no rabbit shows an individual temperature rise of 0.5 °C above its respective control temperature. Thus, the OTC-I has been confirmed to have a good safety profile based on the absence of pyrogens. Indeed, the biocompatibility assessment is required for all medical devices to minimize potential hazards to patients [49]. Animal irritation testing is used to predict whether patient-contacting materials (in this case OTC-I) could cause an irritation response as indicated by oedema, erythema, and eschar formation. In this testing scenario, animals did not show significant erythema and/or edema after intradermal injection of OTC-I extract. Test results showed that the material extracted with non-polar and polar solvents did not irritate the skin of rabbits during the 72-hour observation period. The OTC-I met requirements since the difference between the average test score and the average control score was <1.0 (=0.22), which is in accordance with the normative guidelines ISO 10993-23 [36], and considered non-irritating. Hence, it can be concluded that the tested material does not induce an intradermal reaction. In a study supporting this result, carried out by Radjabov et al., the authors found that the injection solution of collagen does not cause an allergic reaction, does not sensitize the body, and does not cause a pronounced inflammatory reaction, which indicates the safety of its use [50].

Furthermore, acute toxicity of the OTC-I extraction after being injected intravenously to the mice was studied as recommended by ISO 10993-11:2017. The data obtained show that no single mortality was recorded. The mortality effect was said to be one of the observable signs of toxicity due to the failure of several systems, such as the renal and nervous systems [51]. Besides the mortality rate, observation quantitatively through the body weight of before and after also can be used as one of the signs of toxicity. The data obtained with slight body weight reduction is not sufficiently significant to say that the extracted ovine tendon collagen may demonstrate toxicity. The slight reduction in body weight might be because of the decreased appetite of the tested mice or it might not [52]. Thus, clinical changes were further observed to investigate the effect of the extracted ovine tendon collagen. The typical clinical signs and observations, as listed in Annex C International Organisation of Standard, were used as a guideline while observing the behaviour of the mice [38]. No abnormalities detected (NAD) was a short form to conclude that all the mice were normal and healthy. Collagen is a natural protein found in the body and is generally considered safe. However, excessive intake of collagen supplements or injections can potentially lead to toxicity and adverse effects on internal organs. The liver plays a vital role in the metabolism of xenobiotics and endogenous molecules are also said to be a primary site of metabolism that usually appears abnormal if there are any signs of toxicity [53]. This study found that at 50 mg/mL, no animals show any abnormalities in their liver. The kidney, pancreas, lung, heart, and brain were also isolated and observed for any abnormalities. None of the listed organ shows any abnormalities; thus, we could conclude that the extracted OTC-I may not demonstrate toxicity under the ISO 10993-11:2017 guideline [38].

The previous study shows that the fibroblasts are efficiently attached to the non-crosslinked collagen sponge from ovine tendon. It is not only physically in good shape, but the cells can also produce ECM protein [32]. It was also reported in 2016 that collagen type-I can maintain the optical density in day 1 until day 7 after being left in 96-well plates seeded with L-929 mouse fibroblast cells in L-DMEM [54]. In this study, in the L-929 cell line (NCTC clone 929) that appears healthy with no detection of cell lysis or reduction in the number of cell growth, it is believed that the extracted OTC has not demonstrated the toxicity effect. In contrast, the human dermal fibroblast significantly promotes the attachment and proliferation on seeded on the surface of ovine tendon collagen sponge [29]. The L-929 cell line recommended by International Standard Organisation for primary cytotoxicity evaluation is commonly used as the test system in other cytotoxicity studies [55]. With the intention of exposing the OTC-I to the direct wound area, the mutagenicity assessment shall be considered. Collagen matrices derived from porcine pericardium were studied and showed no mutagenic effects had widened the collagen application [56]. The bacterial reverse mutation assay (Ames test) provides a rapid assessment of the mutagenic potential of a compound to cause mutations in the DNA of the test organism [40]. A few strains of *S. typhimurium* and *E. coli* can be used to identify the potential of mutagenic activity such as TA1535, TA100, TA1537, TA97a, TA1538, TA98, TA102, WP2 *uvrA*, and pKM101. The strains also have different primary mechanisms and sensitivity levels. However, the ability to conclude mutagenicity differs according to the required aim [57].

Following the results mentioned above, it can be concluded that OTC-I is highly safe with no infection, skin irritation, allergic reactions, and cytotoxicity. These biocompatibility assessments discovered that OTC-I has no adverse effects in respective studies condition of induced skin sensitisation effect, mutagenic and cytotoxic. Therefore, OTC-I is a potential medical device that is candidate for future wound management. However, vast numbers of experimental animals are still employed to prove the negative results based on cells and tissue of human origin with better significance to human clinical practice to demonstrate their accuracy and reliability [58,59,60]. In summary, our study has provided strong evidence that implantation of OTC-I biomatrix can be used as a feasible and effective therapeutic approach to improve wound healing. This biocompatible OTC-I provides an attractive remedy for wound healing and tissue regeneration; nevertheless, controlled and randomized clinical trials on the implantation of OTC-I are urgently needed to be conducted.

## 5. Conclusions

The safety properties of the OTC-I biomatrix were evaluated as recommended on the biocompatibility testing matrix of ISO 10993-1:2018 that involved in vitro and in vivo coverage, which appears to be a promising, essential result in wound healing and skin regeneration. The negative response of potential toxicity on skin irritation and sensitisation shows that the OTC-I is safe to be used for implantation in wounds. In addition to that, injection of the OTC-I in the pyrogen test approved that the OTC-I does not cause a temperature fluctuation that resembles the effect of the toxicity internally. The data from the in vivo study were strengthened with the parallel outcome of the in vitro testing. The cytotoxicity and genotoxicity effects were also explored in this study, and the result supports the optimistic result from in vivo as expectedly. The mechanical and physicochemical properties of the OTC-I that have been widely investigated over the years are said to have bright potential to be used as a wound-healing treatment when the split skin graph comes to its limit. These safety evaluations on pre-clinical setting help increase the capability of OTC-I to be used as a medical device in the future even though the assessment on the bigger animal is still under question. The OTC-I made from pure collagen has a query on their stability when applied on humans that have exposure to a varied range of temperatures. The aforementioned biological properties of OTC-I revealed that more collagen-based biomatrix scaffolds would reach clinical trials. In conclusion, although further studies are still required, experiments, particularly those using large animals and humans tested, to evaluate the clinical suitability of OTC-I towards humans, to prove that this biomatrix is a strongly promising biomaterial to be used as a scaffold in skin tissue engineering and regenerative medicine, are needed. Further, the implantation test to assess both the local effect (ISO 10993-6) and systemic toxicity (ISO 10993-11) of OTC-I, and the processes of the absorption of OTC-I associated with implantation (ISO 10993-13) were not our primary scope in this study as these were not required by our local authority for medical device registration. However, these would be an extra benefit for future studies.

## Figures and Tables

**Table 1:** Items applied on injection sites during intradermal injection phase.

Site	Test Group	Negative Control Group	Positive Control Group
A	An equal ratio of Freund’s complete adjuvant and normal saline	An equal ratio of Freund’s complete adjuvant and normal saline	An equal ratio of Freund’s complete adjuvant and 80% ethyl alcohol
B	Test item extract only	Normal saline only	0.08% DNCB in 80% ethyl alcohol only
C	Test item extract emulsifies in an equal ratio of Freund’s complete adjuvant and normal saline	Normal saline emulsified in an equal ratio of Freund’s complete adjuvant and normal saline	0.08% DNCB in 80% ethyl alcohol emulsified in an equal ratio of Freund’s complete adjuvant and 80% ethyl alcohol

**Table 2:** Grading scale of cutaneous reaction based on Magnusson and Kligman scale.

Patch Test Reaction	Grading Scale
No visible change	0
Discrete or patchy erythema	1
Moderate and confluent erythema	2
Intense erythema and/or swelling	3

**Table 3:** Grading system for intracutaneous (intradermal) reactions.

Reaction	Description	Score
Eryhtema (E)	Erythema and eschar formation	
	No erythema	0
	Very slight erythema (barely perceptible)	1
	Well-defined erythema	2
	Moderate erythema	3
	Severe erythema (beet-redness) to eschar formation preventing grading of erythema	4
Oedema (O)	Oedema formation	
	No oedema	0
	Very slight oedema (barely perceptible)	1
	Well-defined oedema (edges of the area well-defined by definite raising)	2
	Moderate oedema (raised approximately 1 mm)	3
	Severe oedema (raised more than 1 mm and extending beyond exposure area)	4
Maximal possible score for irritation		8

Other adverse changes at the skin sites shall be recorded and reported.

**Table 4:** Common clinical signs and observations.

Observation	Observed Sign	Involved System (s)
Respiratory	Dyspnoea (abdominal breathing, gasping), apnoea, cyanosis, tachypnoea, nostril discharges	Central nervous system (CNS), pulmonary, cardiac
Motor activities	Decrease/increase somnolence, loss of righting, catalepsy, ataxia, unusual locomotion, prostration, tremors, fasciculation	Central nervous system (CNS), somatomotor, sensory, neuromuscular, autonomic
Convulsion	Clonic, tonic, tonic-clonic, asphyxia, opisthotonos	Central nervous system (CNS), neuromuscular, autonomic, respiratory
Reflexes	Corneal, righting, myotatic, light, startle reflex	Central nervous system (CNS), sensory, autonomic, neuromuscular
Ocular signs	Lacrimation, miosis, mydriasis, exophthalmos, ptosis, opacity, iritis, conjunctivitis, chromodacryorrhea, relaxation of nictitating membrane	Autonomic, irritation
Cardiovascular signs	Bradycardia, tachycardia, arrhythmia, vasodilation, vasoconstriction	Central nervous system (CNS), autonomic, cardiac, pulmonary
Salivation	Excessive	Autonomic
Piloerection	Rough hair	Autonomic
Analgesia	Decrease reaction	Central nervous system (CNS), sensory
Muscle tone	Hypotonia, hypertonia	Autonomic
Gastrointestinal	Soft stool, diarrhoea, emesis, diuresis, rhinorrhoea	Central nervous system (CNS), autonomic, sensory, GI motility, kidney
Skin	Oedema, erythema	Tissue damage, irritation

**Table 5:** Qualitative method for determination the biological reactivity of the L-929 cell line.

Grade	Reactivity	Condition of Cultures
0	None	Discrete intracytoplasmatic granules, no cell lysis, no reduction in cell growth
1	Slight	Not more than 20% of the cells are round, loosely attached, and without intracytoplasmatic granules, or show changes in morphology; occasional lysed cells are present; only slight growth inhibition observation
2	Mild	Not more than 50% of the cells are round, devoid of intracytoplasmatic granules, no extensive cell lysis; not more than 50% growth inhibition observable.
3	Moderate	Not more than 70% of the cell layers contain rounded cells or are lysed; cell layers not completely destroyed, but more than 50% growth inhibition observable.
4	Severe	Nearly complete or complete destruction of the cell layers.

**Table 6:** Dose/final concentration of positive controls per plate for each bacterial strain.

Strains	Without Metabolic Activation	With Metabolic Activation
*S. typhimurium* TA98	4-Nitro-o-phenylenediamine	2-Aminoanthracene
	(2.5 µg/plate/25 µg/mL)	(0.5 µg/plate/5 µg/mL)
*S. typhimurium* TA100	Sodium azide	2-Aminoanthracene
	(5.0 µg/plate/50 µg/mL)	(1.0 µg/plate/10 µg/mL)
*S. typhimurium* TA1535	Sodium azide	2-Aminoanthracene
	(0.5 µg/plate/5 µg/mL)	(2.0 µg/plate/20 µg/mL)
*S. typhimurium* TA1537	9-Aminoacridine	2-Aminoanthracene
	(50 µg/plate/500 µg/mL)	(2.0 µg/plate/20 µg/mL)
*E. coli* WP2 *trp uvrA*	Methyl methanesulfonate	2-Aminoanthracene
	(2.5 µL/plate/25 µL/mL)	(10.0 µg/plate/100 µg/mL)

**Table 7:** Temperature variations recorded (Sham test).

Time (min)		0	30	60
Test Animal Sequence	Initial Weight (kg)/Sex	Main Temperature (°C)		
A050-22-01	3.46/Female	39.2	38.9	39.0
A050-22-02	2.62/Female	39.6	39.6	39.6
A050-22-03	2.92/Female	39.3	39.7	39.7

**Table 8:** Temperature variations recorded in response to the test solution (Main test).

Time (min)	Control Temperature	30	60	90	120	150	180	ΔT
Volume Administered (mL)	Mean Temperature (°C)							
34.6	39.1	39.1	38.9	39.1	39.4	39.3	39.4	0.3
26.2	39.4	39.6	39.5	39.5	39.4	39.6	39.5	0.3
29.2	39.5	39.4	39.2	39.3	39.4	39.3	39.4	0.0

**Table 9:** Cutaneous reaction scores of OTC-I and their corresponding control.

Test Item	Animal Number/ Sex	Subtotal E + O			Mean Score	Mean Score for Solvent Extract/ Solvent Control
		24 ± 2 h Post-Injection	48 ± 2 h Post-Injection	72 ± 2 h Post-Injection		
OTC-I + normal saline	Rabbit 1/Female	0	0	0	0	0
	Rabbit 2/Female	0	0	0	0	
	Rabbit 3/Female	0	0	0	0	
Normal saline (Polar solvent control)	Rabbit 1/Female	0	0	0	0	0
	Rabbit 2/Female	0	0	0	0	
	Rabbit 3/Female	0	0	0	0	
OTC-I + Cottonseed oil	Rabbit 1/Female	1	4	4	0.60	0.29
	Rabbit 2/Female	0	1	0	0.07	
	Rabbit 3/Female	1	1	1	0.20	
Cottonseed oil (Non-polar solvent control)	Rabbit 1/Female	1	1	4	0.20	0.07
	Rabbit 2/Female	0	0	0	0	
	Rabbit 3/Female	0	0	0	0	

**Table 10:** The behavioural changes in Balb/c mice immediately, 4 ± 1 h and to day 3 (72 ± 2 h) after injection.

Treatment Group	Animal ID	Observation				
		Immediately after Injection	(4 ± 1) Hours after Injection	Day 1	Day 2	Day 3
Control	GM006E	NAD	NAD	NAD	NAD	NAD
	GM007E	NAD	NAD	NAD	NAD	NAD
	GM008E	NAD	NAD	NAD	NAD	NAD
	GM009E	NAD	NAD	NAD	NAD	NAD
	GM010E	NAD	NAD	NAD	NAD	NAD
OTC-I	GM004E	NAD	NAD	NAD	NAD	NAD
	GM005E	NAD	NAD	NAD	NAD	NAD
	GM011E	NAD	NAD	NAD	NAD	NAD
	GM012E	NAD	NAD	NAD	NAD	NAD
	GM013E	NAD	NAD	NAD	NAD	NAD

NAD = No abnormalities detected.

**Table 11:** The necropsy findings of Balb/c mice among the group.

Treatment Group	Animal ID	Necropsy Findings
Control	GM006E	No abnormal findings. All organs were normal.
	GM007E	No abnormal findings. All organs were normal.
	GM008E	No abnormal findings. All organs were normal.
	GM009E	No abnormal findings. All organs were normal.
	GM010E	No abnormal findings. All organs were normal.
OTC-I	GM004E	No abnormal findings. All organs were normal.
	GM005E	No abnormal findings. All organs were normal.
	GM011E	No abnormal findings. All organs were normal.
	GM012E	No abnormal findings. All organs were normal.
	GM013E	No abnormal findings. All organs were normal.

**Table 12:** Rreactivity grades of the extracted OTC-I at 100% concentration and control item.

Groups	0 h			24 h		
	Well A	Well B	Well C	Well A	Well B	Well C
100% OTC-I	0	0	0	0	0	0
Blank	0	0	0	0	0	0
Negative Control	0	0	0	0	0	0
Positive Control	0	0	0	4	4	4

## Data Availability

The data presented in this study are available on request from the corresponding author.
